# 2326. SARS-CoV-2 PCR Cycle Threshold Trends in Immunocompromised Patients and Implications for Isolation Precautions

**DOI:** 10.1093/ofid/ofad500.1948

**Published:** 2023-11-27

**Authors:** Courtney E Harris, Vineeta Vaidya, Michael Klompas, Chanu Rhee, Lindsey R Baden, Meghan Baker

**Affiliations:** Brigham & Women's Hospital, Boston, Massachusetts; Brigham and Women's Hospital, Boston, Massachusetts; Harvard Medical School and Harvard Pilgrim Health Care Institute, Boston, Massachusetts; Brigham and Women's Hospital, Boston, Massachusetts; Brigham and Women's Hospital, Boston, Massachusetts; Brigham and Women's Hospital, Boston, Massachusetts

## Abstract

**Background:**

RT-PCR assays for SARS-CoV-2 produce a cycle threshold (Ct) value inversely proportional to the amount of virus detected. Ct values are sometimes used as proxies for infectivity to inform duration of precautions, but trends in Ct values vary between patient populations. We sought to better characterize the potential duration of infectivity using Ct values in immunocompromised patients and how it may vary between different kinds of immunocompromising conditions.

**Methods:**

We performed a single-center, retrospective study of all inpatients or outpatients with solid or hematologic malignancies and a SARS-CoV-2 PCR test with available Ct counts between 12/1/2021 and 9/30/2022 (Omicron predominant period). Patient testing, immunocompromised status, and recent medications were analyzed. Patients with a positive PCR test with Ct value ≤33 were included if they had at least 2 tests within 30 days. PCR tests with Ct values of >33 were considered negative (non-infectious).

**Results:**

398 patients with 399 episodes of SARS-CoV-2 infection were evaluated. 210 (53%) patients had a solid organ malignancy, and 189 (47%) had a hematologic malignancy; in the latter group, 138 (73%) had B-cell depleting therapy or Bruton tyrosine kinase inhibitor (BTKi) therapy in the 6 months preceding their first positive test. SARS-CoV-2 PCRs remained positive with Ct values ≤33 for ≥20 days after a first positive test in 46/189 (24%) patients with hematologic malignancies versus 15/210 (2%) with solid organ malignancies. In the subset who received B-cell depleting or BTKi therapy, 37/138 (27%) tested positive at ≥20 days compared to 9/51 (18%) of those who did not [**Figure 1**]. Testing remained positive for ≥30 days in 28/189 (15%) patients with hematologic malignancies vs 2/210 (1%) patients with solid organ malignancies [**Table 1]**.

**Figure:**
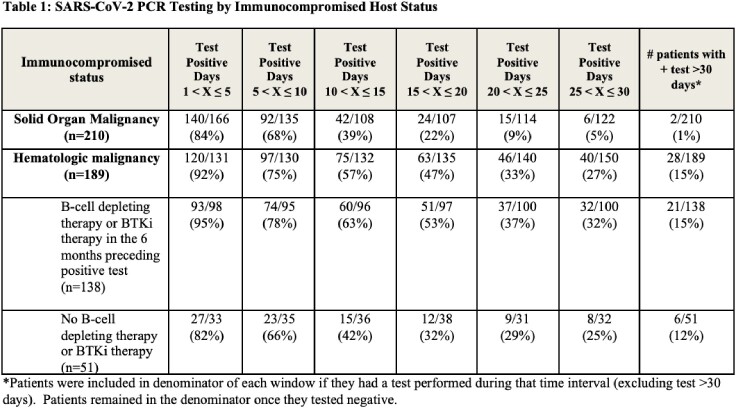

*Patients were included in the denominator of each window if they had a test performed during that time interval (excluding tests>30 days). Patients remained in the denominator once they tested negative.

**Figure:**
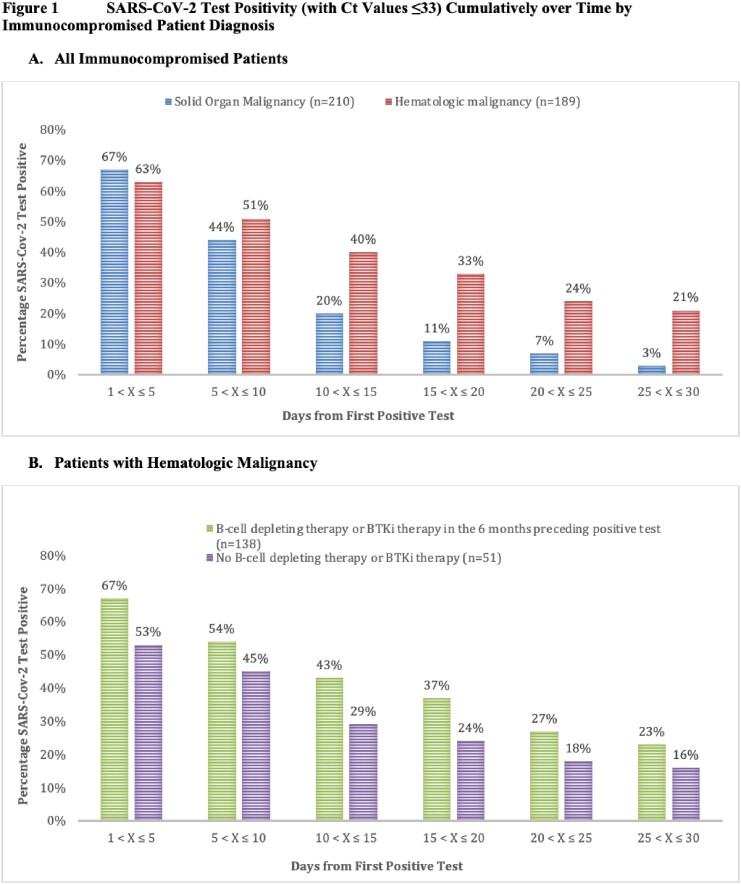

A. All Immunocompromised Patients B. Patients with Hematologic Malignancy

**Conclusion:**

Patients with hematologic malignancies, specifically those with B-cell depleting or BTKi therapy, were much more likely to test positive with Ct values in the potentially infectious range for prolonged periods compared to those with solid organ malignancy. Time-based strategies for discontinuing precautions should consider not only immunocompromised status but underlying conditions. Test-based clearance may be preferable for patients with hematologic malignancies.

**Disclosures:**

**Courtney E. Harris, MD**, Dynamed: Advisor/Consultant **Michael Klompas, MD, MPH**, UpToDate, Inc.: Royalties for chapters on pneumonia **Chanu Rhee, MD, MPH**, Cytovale: Advisor/Consultant|Pfizer: Advisor/Consultant|UpToDate, Inc.: Honoraria

